# The Great International Exhibition of 1862

**Published:** 1861-09

**Authors:** 


THE GREAT INTERNATIONAL EXHIBITION OF 1862.—The Queen has appointed the following gentlemen to act as members of a committee on surgical instruments and appliances for the approaching Exhibition: William Lawrence, Joseph Henry Green, Moncrieff Arnott, John F. South, Cæsar H. Hawkins, James Luke, F. Seymour Huden, and James Paget.

These gentlemen all know what a good knife is, and how to wield it upon the living subject.

